# Genomewide identification of pheromone-targeted transcription in fission yeast

**DOI:** 10.1186/1471-2164-7-303

**Published:** 2006-11-30

**Authors:** Yongtao Xue-Franzén, Søren Kjærulff, Christian Holmberg, Anthony Wright, Olaf Nielsen

**Affiliations:** 1 School of Life Sciences, Södertörns Högskola, SE-141 89 Huddinge, Sweden and Department of Bioscience and Nutrition, Karolinska Institutet, SE 141 57 Huddinge, Sweden; 2 Department of Molecular Biology, University of Copenhagen, DK-1353 Copenhagen K, Denmark

## Abstract

**Background:**

Fission yeast cells undergo sexual differentiation in response to nitrogen starvation. In this process haploid M and P cells first mate to form diploid zygotes, which then enter meiosis and sporulate. Prior to mating, M and P cells communicate with diffusible mating pheromones that activate a signal transduction pathway in the opposite cell type. The pheromone signalling orchestrates mating and is also required for entry into meiosis.

**Results:**

Here we use DNA microarrays to identify genes that are induced by M-factor in P cells and by P-factor in M-cells. The use of a *cyr1 *genetic background allowed us to study pheromone signalling independently of nitrogen starvation. We identified a total of 163 genes that were consistently induced more than two-fold by pheromone stimulation. Gene disruption experiments demonstrated the involvement of newly discovered pheromone-induced genes in the differentiation process. We have mapped Gene Ontology (GO) categories specifically associated with pheromone induction. A direct comparison of the M- and P-factor induced expression pattern allowed us to identify cell-type specific transcripts, including three new M-specific genes and one new P-specific gene.

**Conclusion:**

We found that the pheromone response was very similar in M and P cells. Surprisingly, pheromone control extended to genes fulfilling their function well beyond the point of entry into meiosis, including numerous genes required for meiotic recombination. Our results suggest that the Ste11 transcription factor is responsible for the majority of pheromone-induced transcription. Finally, most cell-type specific genes now appear to be identified in fission yeast.

## Background

The fission yeast *Schizosaccharomyces pombe *provides an attractive model system for studying cellular differentiation processes in response to environmental cues. It is a unicellular, haploid organism with a conventionally organized cell cycle [1], and it is easily subjected to genetic analysis [2]. Furthermore, since its entire genome sequence is known [3], genome-wide analysis tools can be readily applied.

**Figure 1:**
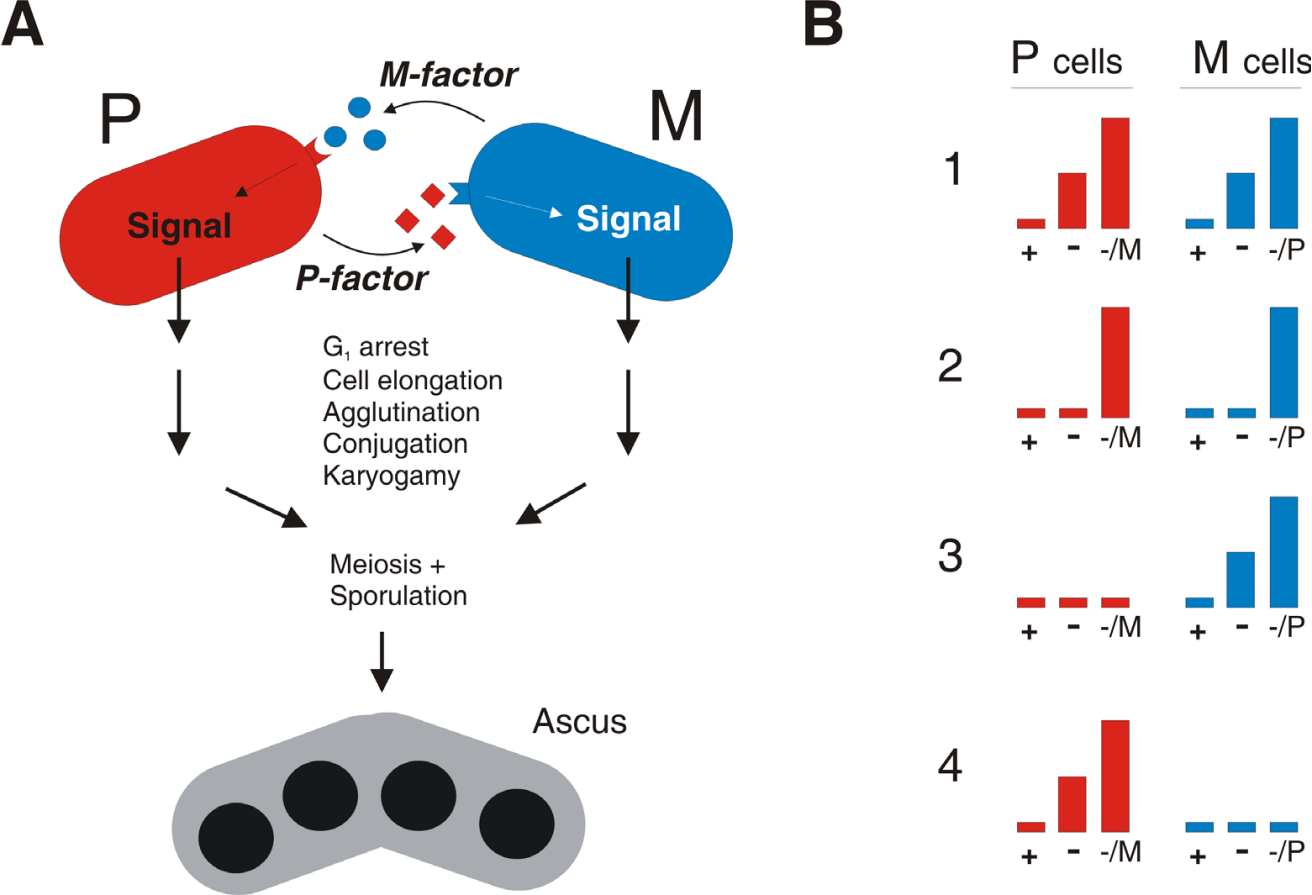
**Pheromone-induced transcription in *S. pombe***. A. Diagram of pheromone communication. Prior to mating, M cells and P cells secrete the two pheromones, P-factor (squares) and M-factor (circles) that can bind to specific receptors present on the surface of the opposite cell type. This causes activation of an intracellular signalling pathway that prepares the cells for mating. Following successful conjugation, continued pheromone stimulation allows entry into meiosis and sporulation. B. Types of pheromone regulated transcription. Bar diagrams symbolizing the transcription levels in cells growing in the presence of nitrogen (+), starved for nitrogen (-) or starved for nitrogen in the presence of M-factor (-/M) or P-factor (-/P). The left panel illustrates the expression pattern in P cells, while the right panel shows the pattern in M cells. The following types of genes are depicted: (1) a gene that is induced by nitrogen starvation and further boosted by pheromone signalling, (2) a gene that has an absolute requirement for both nitrogen starvation and pheromone signalling, (3) an M-specific gene, (4) a P-specific gene.

In response to nitrogen starvation fission yeast cells undergo sexual differentiation. In this process haploid P and M cells first mate to form dipoid zygotes [4], which then undergo meiosis and sporulation [5]. Nitrogen starvation leads to the establishment of a pheromone signalling system by which M and P cells communicate with each other (see Figure 1A), causing growth arrest in the G_1 _phase of the cell cycle. Pairs of G_1 _arrested M- and P- cells subsequently agglutinate, mate, and undergo karyogamy, after which the resulting diploid zygotes enter meiosis and finally sporulate.

The HMG-box transcription factor Ste11 is a master regulator of the sexual differentiation programme [6]. Upon starvation, Ste11 activates numerous genes required for mating and meiosis, including the *mat *genes specifying cellular mating type, many genes encoding components of the pheromone communication system, and *mei2 *that encodes the ultimate activator of meiosis. Ste11 binds to one or several Ste11-sites (AACAAAGAAA) present in the promoter regions of the genes it directly controls.

The two pheromones are small, secreted peptides that bind to and activate cell-type specific 7TM receptors on the cell surface (Mam2 in M cells and Map3 in P cells), thereby activating the receptor-coupled G-protein, Gpa1. This stimulates a MAP kinase cascade, consisting of Byr2 (a MAP3K), Byr1 (a MAP2K) and Spk1 (a MAPK). The pheromone response, which is shared by the two cell types, activates genes required at various steps throughout the differentiation process, with entry into meiosis being the most downstream event reported to rely on the signal [7].

Several lines of evidence suggest that Ste11 is also the transcription factor activated by pheromone signalling. Pheromone-controlled genes often have Ste11-boxes in their promoter regions, and hyper-activation of the MAP2K Byr2 causes constitutive activation of Ste11 [8]. Spk1 can in fact directly phosphorylate Ste11 on threonines 305 and 317 *in vitro*, and mutational analysis of these residues *in vivo *is consistent with a direct activation mechanism [8]. However, since Ste11 is absolutely required for the expression of many components in the response pathway, it is difficult to evaluate whether pheromone signalling can stimulate transcription in the absence of Ste11.

The analysis of pheromone-controlled transcription is further complicated by the nutritional control over the differentiation pathway. In order to make them respond to mating pheromones, it is necessary to starve cells for nitrogen, which in itself causes dramatic changes in the expression of many genes [9,28]. Furthermore, the transcriptional responses to nitrogen starvation and pheromone stimulation are partially overlapping, and Ste11-controlled genes appear to be induced to varying degrees by both these environmental cues (Figure 1B). Some genes, such as *ste6 *[10] are already strongly induced by nitrogen starvation and become further enhanced by pheromone (Figure 1B, panel 1). Expression of other genes like *fus1 *[11] has an absolute requirement for both nitrogen starvation and pheromone signalling (Figure 1B, panel 2). Genetic analysis has shown that *byr2*, *byr1 *and *ste11 *are required for induction of pheromone controlled genes by nitrogen starvation [12], suggesting that this signal is also conveyed by the pheromone-responding MAPK pathway.

An additional layer of regulation is conferred by cellular mating type. Whilst most pheromone-controlled genes appear to be equally expressed in the two cell types, a small subset is either M-specific (Figure 1B, panel 3) or P-specific (Figure 1B, panel 4). These genes are involved in synthesis and processing of the cell-type specific extracellular components of the pheromone-communication system, i.e. the pheromones and receptors. Their expression is controlled by the allele present at the *mat1 *locus of the cell [13]. In M cells the *mat1-Mc *gene causes activation of M-specific genes, while the *mat1-Pc *gene activates P-specific genes in P cells. Mat1-Mc is an HMG-box protein that appears to recruit Ste11 to a weaker version of its binding site (AACAAAGA) present in the promoter region of M-specific genes [14]. The mechanism by which Mat1-Pc activates the P-specific genes is less clear. Genetic analysis suggests that it cooperates with the Map1 protein, a putative transcription factor of the MADS-box family [15,16].

In the present study we use genomic microarrays to identify pheromone-induced genes in fission yeast. In order to discriminate pheromone-induction from the effect of nitrogen starvation, we utilize the fact that *cyr1 *strains defective in cAMP production are derepressed for mating activities, and hence respond to pheromone without being starved [17]. We have identified classes of genes induced by M-factor in P cells and genes induced by P-factor in M-cells. As expected, these two groups of genes were highly overlapping. Moreover, a direct comparison of M and P cells allowed us to identify several new genes that are either M- or P-specific. Our results support the notion that Ste11 is the major transcription factor bringing about pheromone-stimulated transcription.

## Results

### Identification of genes induced by M- and P-factor

In order to characterize the changes in transcription profiles caused by mating pheromone stimulation, we performed two different experiments; one where we added M-factor to *h*^+^*cyr1 *cells, and a second adding P-factor to *h*^-^*cyr1 sxa2 *cells. The *cyr1 *mutation was included in both strains in order to enable pheromone stimulation without first subjecting the cells to nitrogen starvation [18]. Furthermore, the *h*^- ^strain was inactivated in the *sxa2 *gene, which encodes a very potent P-factor degrading protease [19].

We took samples for RNA preparation after 2, 4 and 6 hours from both cultures. Total RNA from each time point was hybridised to genomic arrays using the 0 hour time point from the same mating type as a reference. The complete data set is available from the Gene Expression Omnibus database (see Materials and Methods). Analysis of the data showed that pheromone treatment caused dramatic induction of a relatively large number of genes. K-means cluster analysis of these genes revealed that most genes were already induced after 2 hours of pheromone stimulation, and that their expression remained high for the duration of the experiment (data not shown), suggesting that pheromone induction occurred rapidly. For each pheromone, we therefore identified genes that were consistently induced more than twofold at all three time-points after pheromone addition. Using these criteria, we identified 120 M-factor induced genes and 93 P-factor induced genes [see Additional file 1]. A direct comparison revealed that most pheromone-induced genes were expressed at similar levels in untreated *h*^+ ^and *h*^- ^cells [see Additional file 1].

**Figure 2:**
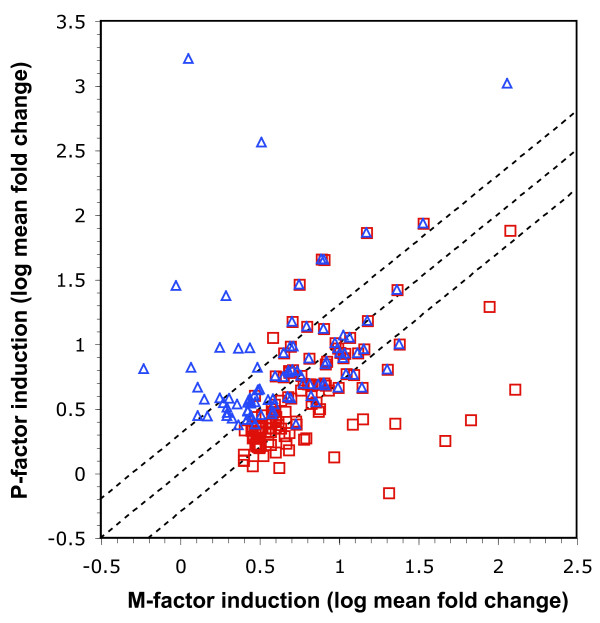
**Comparison of genes induced by M-factor and P-factor**. Log_10 _mean fold change values for P-factor and M-factor induction are compared in a scatter plot. Values are plotted separately for genes persistently induced (≥ 2-fold) by M-factor (red squares) and P-factor (blue triangles). The dotted lines show the position of genes that are equally affected by each pheromone (middle line) as well as genes for which induction by M-factor is 2-fold that observed for P-factor (lower line) and vice versa (upper line).

Comparison of the sets of M-factor and P-factor induced gene lists showed that they were highly overlapping, containing many genes in common. This is consistent with the fact that the pheromone response pathway is common to both mating types, and most of the proteins required in the differentiation pathway perform similar functions in the two cell types. To analyse the similarity of the responses further we compared the M-factor and P-factor induced changes for both sets of induced genes. Figure 2 shows that the fold change values for M- and P- treatment are highly correlated for most genes. Most of the minority of genes that are less well correlated still show a tendency to induction under both conditions.

### Pheromone-induced genes

**Figure 3:**
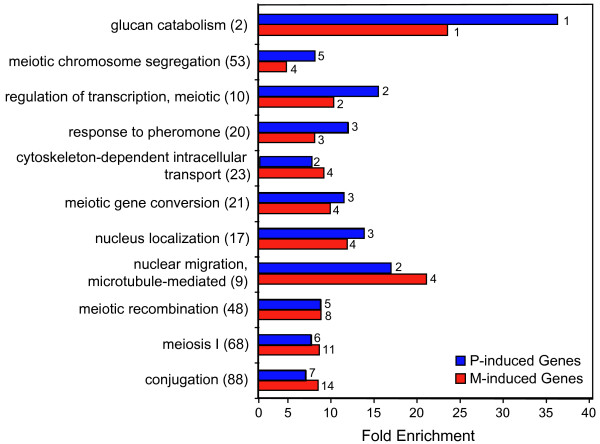
**Representative Gene Ontology (GO) categories selectively associated with the groups of genes that are induced by M-factor and P-factor**. The bar chart shows the level of enrichment for the selected categories compared to their frequency in the genome. All enrichment levels are significance at the 95% confidence level or higher. Numbers in parenthesis show the number of gene in each GO category. The numbers associated with each bar in the chart show the number of genes in the M-factor and P-factor induced groups of genes that are annotated with each GO category. The groups of genes induced by M-factor (red bars) and P-factor (blue bars) contained genes from the groups studied in Figure 2 where the mean fold change value for the different time points was ≥ 2 (SD < 85%) and the change in expression was statistically significant (*p *≤ 0.01).

The list identified by our array experiments contained genes such as *bqt1*, *fus1 *and *rep1 *previously demonstrated to be pheromone controlled, but also many new genes (see Table 1). To investigate the function of the pheromone induced genes further, we identified Gene Ontolology (GO) categories that were significantly associated with M-factor and P-factor induced genes (Figure 3).

As expected, genes involved in *conjugation *were enriched in both M-factor and P-factor induced genes. In addition, we found genes such as *spk1*,*ste4 *and *ste11 *encoding proteins directly involved in *response to pheromone*.

Other functions, specifically involved in the morphological alterations observed during mating, were also enriched among the pheromone-induced genes. These included *glucan catabolism*, *cytoskeleton-dependent intracellular transport*, and *microtubule-mediated nuclear migration *and *nucleus localization*. Presumably, the induction by pheromone of a specific subset of genes involved in these processes redirects them to accommodate their specific behaviour during differentiation.

The *mat1-Pm *and *mat1-Mm *genes required for entry into meiosis are the most downstream functions so far reported to be pheromone controlled [7]. It was therefore of interest to find gene categories performing specific meiotic functions that lie further downstream (*regulation of meiotic transcription*, *meiosis I *and *meiotic chromosome segregation*) among the up-regulated genes. Genes in these categories include the meiotic master regulator *mei2 *and the meiotic forkhead transcription factor *mei4 *but our results also identify new potential regulators such as the putative forkhead transcription factor (*SPAP14E8.02*). Finally, a relatively large group of genes belonging to the categories *meiotic recombination *and *meiotic gene conversion *were found to be pheromone induced. These include *dmc1*, *eme1*, *rad25*, *rad32, rec10*, *rec12*, *rec25*, *rec27*, *rti1 *and *swi5*.

### Validation and functional characterization

**Figure 4:**
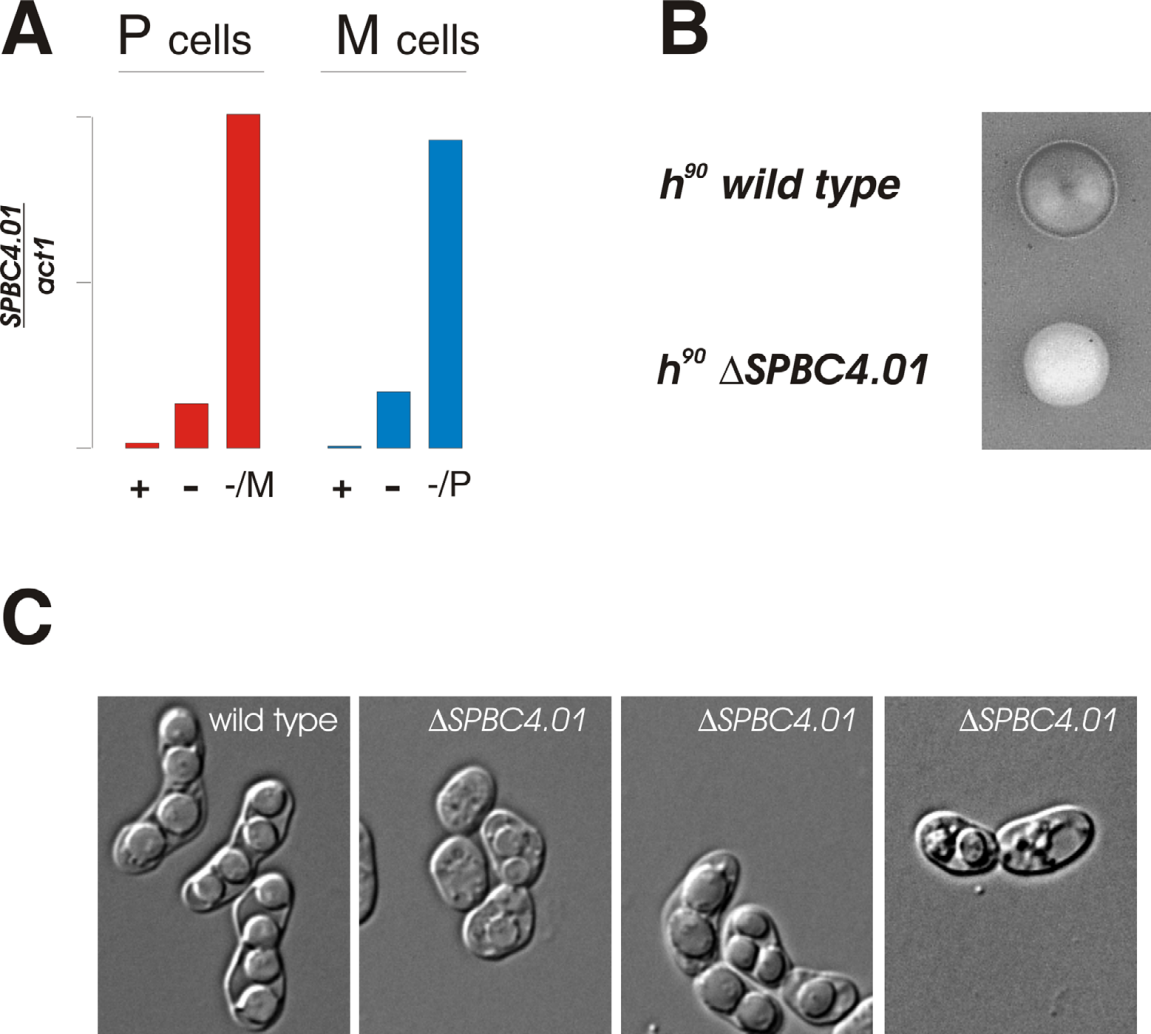
**Analysis of the pheromone-induced gene *SPBC4.01***. A. Expression of *SPBC4.01 *in P cells (left half, red bars) and M cells (right half, blue bars). Cells were either growing in medium with nitrogen (+), without nitrogen (-), or without nitrogen in the presence of M factor (-/M) or P-factor (-/P). At the different growth conditions, the level of *SPBC4.01 *RNA was quantitated relative to *act1 *RNA by real time PCR. B. Sporulation defect of Δ*SPBC4.01*cells. The picture shows iodine staining of *h*^90 ^wild type and *h*^90 ^Δ*SPBC4.01 *colonies grown on the same MSA plate. C. Micrographs of *h*^90 ^wild type and *h*^90 ^Δ*SPBC4.01 *cells showing examples of abnormal mating and sporulation in the latter.

To confirm that the newly identified genes were actually pheromone controlled, we performed real-time RCR analysis on several of them (Figure 4 and data not shown). In order to do this, we did the pheromone stimulation with *cyr1*^+ ^cells that were also starved for nitrogen. This allowed us not only to validate the results in a different genetic background, but also to evaluate the inducibility by nitrogen starvation of the pheromone-induced genes. As an example, Figure 4A shows the analysis of the gene *SPBC4.01*, which was strongly induced by both pheromones in the array experiment. The real-time PCR data showed that this gene was already induced by nitrogen starvation, and confirmed that its expression was further elevated by pheromone addition in both mating types, leading to a more than 50 fold induction in total.

*SPBC4.01 *encodes a sequence orphan with no homologs in other organisms. In order to examine whether the gene was required for sexual differentiation, we disrupted the gene. As judged by iodine starvation, elimination of *SPBC4.01 *indeed caused a dramatic defect in the ability of cells to undergo sporulation (Figure 4B), as both the rate of entry into differentiation and the final level of sporulation were strongly reduced. Microscopic examination revealed that the disrupted strain was defective in both cell fusion and spore formation (Figure 4C). In contrast, loss *of SPBC4.01 *did not seem to affect mitotic cell growth severely (data not shown). In conclusion, the array data allowed us to identify novel pheromone-induced genes involved in sexual differentiation. Furthermore, the real-time PCR confirmed their regulation by pheromone, and demonstrated that such genes could also be induced by nitrogen starvation alone.

### Pheromone controlled genes have an elevated occurrence of putative Ste11 binding sites

**Figure 5:**
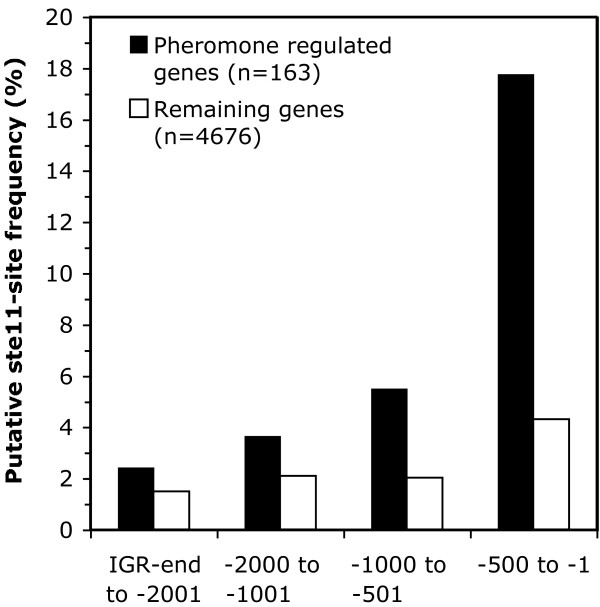
**Enhanced frequency of putative binding sites in pheromone-induced genes**. The bar diagram shows the frequency of genes containing putative Ste11 sites in pheromone-induced genes (closed bars) compared to the remaining genes in the genome (open bars). The number of genes in each set is shown in parenthesis. Perfect matches to a Ste11-binding-site matrix (see Materials and Methods) were counted at different intervals within the inter-genic regions (IGR) upstream of the initiation codon. The group of pheromone-induced genes contains the genes studied in Figure 2 that were induced by either M-factor and/or P-factor. The group of "remaining genes" was created by subtracting the pheromone-induced genes from the list of all *S. pombe *genes.

As outlined above, several lines of evidence suggest that Ste11 is the transcription factor activated in response to pheromone signalling. We therefore asked whether the pheromone-controlled genes identified by our array study had Ste11 binding sites in their promoter regions. The number of putative Ste11 binding sites as well as the sequence and position of the best putative site are provided in Additional file 1. Figure 5 shows that Ste11 binding sites within 500 bp upstream of the ATG start codon are about four-fold more frequent in pheromone-regulated genes than in other genes. Ste11 sites are also found further upstream but their frequency gradually drops towards a background level of about 2%. This background level is also seen for the remaining genes in the genome that are not included in our pheromone-induced set. For these genes the background level is fairly constant showing only a slight increase in the -500 to -1 region. Thus the increase in the frequency of Ste11 sites in the pheromone-induced genes appears to be specific. We conclude that the frequency of Ste11 binding sites is highly enriched in the promoters of pheromone-regulated genes, consistent with the notion that Ste11 is the major transcriptional regulator involved in their induction.

### Identification of cell-type specific genes

As mentioned in the introduction, the M- and P-specific genes controlled by the *mat1 *locus are also induced by pheromone. We therefore sought to identify cell-type specific genes by looking for transcripts that were differentially induced by the two mating pheromones. We hybridised the two RNA preparations obtained after 6 hours of M-factor and P-factor stimulation to the same array, and selected transcripts that had a more than 2 fold difference in expression levels. By these criteria we identified 8- M-specific and 4 P-specific genes (see below). We confirmed that these genes were selectively induced by either M-factor or P-factor in the array time course described above (data not shown). In Table 2 we have summarized current knowledge on functions and expression patterns of the cell-type specific genes in fission yeast.

### M-specific genes

**Figure 6:**
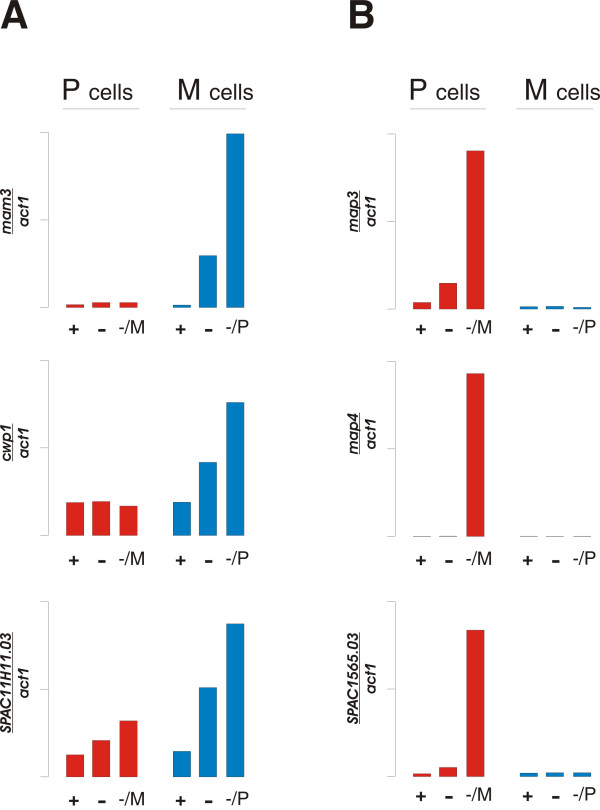
**Expression of cell-type specific genes**. The expression relative to *act1 *was monitored by real time PCR under different growth conditions as described in Figure 4. A. M-specific genes: *mam3 *(upper panel), *cwp1 *(middle panel) and *SPAC11H11.03 *(lower panel). B. P-specific genes: *map3 *(upper panel), *map4 *(middle panel) and *SPAC1565.03 *(lower panel).

The M-specific transcripts included 5 genes previously described to encode M-specific mating functions (*mat1-Mc*, *sxa2*, *mam1, mam2 *and *mam4*); the remaining known M-specific genes (*mfm1-*3 and *mat1-Mm*) are all very short and are not present on the arrays we used. Three new genes (*mam3, cwp1 *and *11H11.03*) were identified, and we confirmed all three to be M-specific by real-time PCR on RNA from *cyr1*^+ ^control cells (Figure 6A).

The first gene, *mam3*, is annotated to encode the M cell agglutinin [20], and hence, its expression was expected to be M-cell specific. The second gene, *cwp1 *encodes the α-subunit of the enzyme geranyl-gernyl transferase I (GGTase I) [21], and its induction by M-factor presumably reflects its role in farnesylation of M-factor [22]. Analogous to the *cwg2 *gene encoding the β-subunit of GGTase I [21], we presume that *cwp1 *is an essential gene, and we have not evaluated its involvement in M-factor production. Consistent with a role of *cwp1 *in other cellular processes, its expression does not have an absolute requirement for M-specific pheromone stimulation (Figure 6A).

The third gene, *11H11.03*, encodes a hypothetical protein that is conserved in eukaryotes. Interestingly, this gene is divergently transcribed from the same promoter region as the M-specific gene *mam2*, and we speculate that the two genes are co-regulated. This gene is also expressed at a relatively high level in P-cells (Figure 6A), and we have not analysed its involvement in mating activities of M-cells.

With these findings, the total number of known M-specific genes is 12. In Table 3 we have aligned their promoter sequences upstream of their Ste11-box. Except for *mam4 *all the M-specific genes contain imperfect Ste11-boxes (AACAAAG) to which Ste11 only binds in the presence of Mat1-Mc [14]. *mam4 *contains a perfect Ste11-box (AACAAAGAAA) to which Ste11 can bind on its own and consistent with this *mam4 *is not strictly M-specific, as it is expressed in both cell types, albeit 2–3 fold higher in M cells [[23], Additional file 1]. Similar to the strict M-specific genes, *mam4 *also harbours an M-box (a Mat1-Mc binding site) 14–26 bp upstream of the Ste11-box (Table 3), which presumably boosts its expression in M cells.

### P-specific genes

Apart from the two mating-type genes *mat1-Pc *and *mat1-Pm *(which are not present on our arrays) only two P-specific genes have been described, namely *map2 *and *map3*, encoding, respectively P-factor and the receptor for M-factor. Both these genes were selectively induced by M-factor in our arrays (Figure 6B, Table 2 and data not shown). The *map4 *gene encoding P-cell specific agglutinin [24] was not identified by our array approach, but real-time PCR analysis showed that its expression indeed was P-specific (Figure 6B). The two additional P-specific genes identified in the arrays (*SPAC1565.03 *and *SPAC3F10.09*), were analysed further. *SPAC1565.03 *was confirmed to be P-specific by real-time PCR analysis (Figure 6B), and hence represents a new bona fide P-specific gene. However, disruption of *SPAC1565.03 *had no obvious effect on mating of P-cells (data not shown), leaving the reason for its P-specific regulation unexplained.

**Figure 7:**
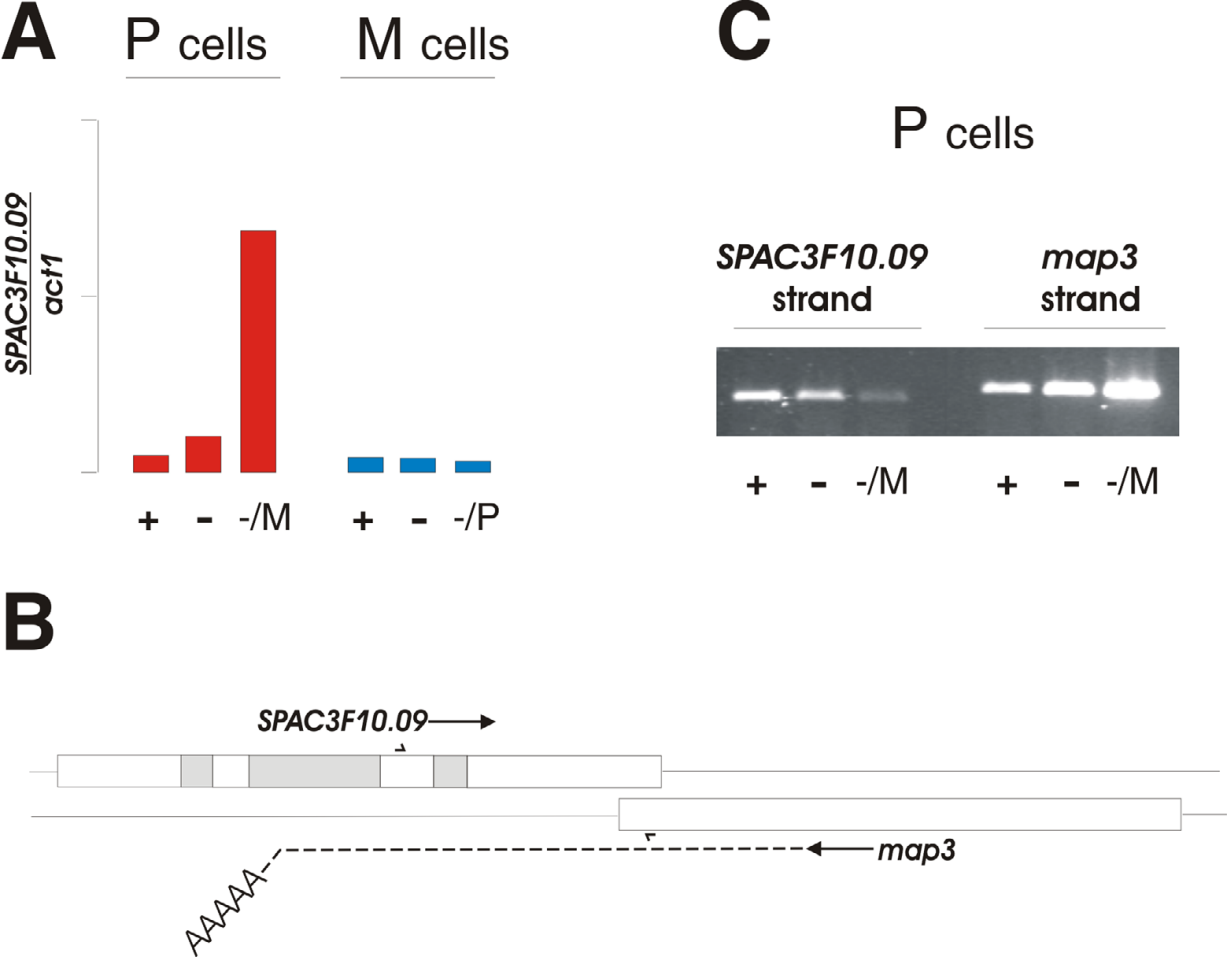
**Analysis of *SPAC3F10.09 *expression**. A. Expression profile of *SPAC3F10.09 *relative to *act1 *(see Figure 4 for experimental details). B. Schematic representation of the chromosomal region encompassing *SPAC3F10.09 *and *map3*. The open reading frames of the two genes are shown as boxes, with arrows after the gene names indicating the direction of transcription. The three introns in *SPAC3F10.09 *are shown in grey. The position of the two *SPAC3F10.09 *specific primers are indicated with half-arrows. The position of the mapped 3'polyA tail of the *map3 *transcript in the second intron of *SPAC3F10.09 *is at the bend of the dotted line. C. Strand-specific RT PCR analysis of *SPAC3F10.09 *showing expression from, respectively, the *SPAC3F10.09 *strand (left half) or the *map3 *strand (right half) on RNA from *h*^+ ^cells under the growth conditions indicated below.

The other P-specific gene, *SPAC3F10.09*, represented a puzzle. Its sequence suggests that it encodes an enzyme homologous to *S. cerevisiae HIS6*, and hence it is likely to be involved in histidine biosynthesis; yet real-time PCR analysis confirmed that its expression was highly induced by pheromone in a P-cell specific manner (Figure 7A). We therefore sought alternative explanations for its apparent P-cell specific expression pattern. We noticed that this gene is partially overlapping with the P-specific gene *map3 *(see Figure 7B). The two genes are convergently transcribed, and their reading frames overlap by 27 codons. Hence, we speculated that the array spots for *SPAC3F10.09 *might have picked up the *map3*-specific signal from the opposite strand. In order to test this hypothesis, we did strand-specific RT-PCR with the same two *SPAC3F10.09 *primers that were used to generate the PCR product constituting the array spots (Figure 7C). Indeed, the P-specific, pheromone-induced transcript originates from the *map3*-specific strand. The *SPAC3F10.09 *specific transcript from the opposite strand gives rise to a somewhat smaller PCR product due to the splicing out of the 66 bases long third intron in the gene (see Figure 7C). Curiously, this transcript seemed to be down-regulated when *map3 *was induced by pheromone, suggesting that strong transcription in the opposite direction can negatively affect gene expression. Consistent with the array data, we mapped the 3'-end of the *map3 *mRNA to the sequence 5'-ACATCCGAGA-3' in the second intron of *SPAC3F10.09 *(see Figure 7B).

## Discussion

In fission yeast, nitrogen starvation causes induction of gene expression by at least three different signalling pathways. First, it leads to a modest reduction in cellular cAMP, which in turn down regulates the glucose sensing Pka1 protein kinase that directly represses transcription of genes such as *fbp1 *and *ste11 *[25]. Secondly, nitrogen starvation activates the Spc1/Sty1 stress response pathway [26], which causes induction of the *atf1 *transcription factor that also controls *ste11 *[27]. Finally, genetic analysis suggests that the pheromone-responding MAPK pathway conveys the signal of nitrogen starvation to pheromone controlled promoters [12].

In the present study we have used a *cyr1 *genetic background to separate pheromone-induced transcription from the general effects of nitrogen starvation. Consequently, we found that pheromone stimulation of *cyr1 *cells caused a much less dramatic change in expression profiles than nitrogen starvation of wild-type cells [9,28]. By this approach we have identified a total of 163 genes that were more than two-fold induced by pheromone stimulation, including several genes previously reported to be pheromone controlled.

Among the pheromone-induced genes we found the cAMP degrading phosphodiesterase Cgs2, suggesting that pheromone signalling enforces the reduction in cAMP caused by nitrogen starvation [29]. This gene was also shown to be induced by activation of the Spc1/Sty1 stress response pathway [30], suggesting that *cgs2 *constitutes a junction for cross talk between different starvation sensing pathways. When cAMP levels drop, the APC/C E3 ubiquitin ligase becomes activated [31], and this causes G1 arrest *via *degradation of the S-phase activating cyclin Cig2 [32]. Consistent with an increased APC/C activity during differentiation, we found *apc2 *encoding a subunit of APC/C among the pheromone-induced genes.

Our identification of components in the pheromone signal transduction pathway (*spk1*,*ste4 *and *ste11*) suggests that initial pheromone stimulation triggers a positive feedback loop enforcing the response. Furthermore, the *whi5 *gene, whose counterpart in *S. cerevisiae *encodes an inhibitor of transcriptional activities required for vegetative S-phase entry [33,34], was among the induced genes, suggesting that pheromone signalling inhibits vegetative growth. Taken together these observations underscore the autocatalytic and irreversible nature of the developmental switch.

On the other hand, functions that dampen the pheromone response were also identified. Thus, the *msa1 *gene encoding an RNA-binding protein that negatively regulates differentiation specific transcripts [35], and *rgs1 *coding for a GTPase that down-regulates the receptor-coupled G-protein Gpa1 [36] were both pheromone induced, stressing the importance of a tight modulation of the response during differentiation.

The results presented here are consistent with Ste11 being the transcription factor activated by pheromone signalling. The pheromone-regulated genes in Figure 5 contain perfect matches to a matrix constructed from known sites and they together account for about 30% of the pheromone-induced transcripts. The remaining pheromone-regulated genes almost invariably contain more diverged derivatives of the Ste11-binding site, often in multiple copies. It is thus likely that Ste11 regulates the majority of pheromone-regulated genes, perhaps in some cases via co-operative binding to a number of relatively weak binding sites as previously reported [14]. It is, however, likely that some pheromone-regulated genes are indirectly induced by other transcription factors, either alone or in combination with Ste11. Indeed, several transcription factors such as *rep1 *and *mei4 *are found within the group of pheromone-induced genes.

The *isp6 *gene, which we found to be pheromone induced, was actually reported to be induced by nitrogen starvation independently of *ste11 *[37], and it does not have an obvious Ste11-box in its promoter region [see Additional file 1]. The cAMP pathway negatively regulates *isp6 *transcription [37], and presumably the pheromone effect on this gene is caused indirectly by cross talk with other signalling pathways as discussed above.

The *cig2 *gene, encoding an S-phase activating cyclin, is specifically expressed in the G_1 _phase of the cell cycle [38], and hence its induction by pheromone could be an indirect consequence of pheromone-mediated G_1 _arrest However, the *cig2 *promoter contains a perfect Ste11-box [see Additional file 1], suggesting that Ste11 and the pheromone pathway may also directly control this gene. The regulation of *cig2 *represents a paradox: Transcription of the gene is on the one hand induced by pheromone, yet the same signal also mediates proteolysis of the Cig2 protein [32]. Presumably this regulation reflects the fact that Cig2 needs to be down-regulated in order to activate the differentiation pathway, but then Cig2 is needed again soon after for activation of pre-meiotic S-phase [39].

By comparing the effects of M- and P-factor stimulation we were able to identify P-and M-specific genes. Given the extensive hunt over many years for cell-type specific mutants, it was no surprise that we only found very few new genes, and an important conclusion of this work is that the list in Table 2 can be regarded, as close to being complete. The larger number of M-specific genes largely reflects the more elaborate synthesis of M-factor relative to P-factor.

Our results confirm the proposed mechanism of M-specific gene activation [14], where the Mat1-Mc protein both recruits Ste11 to a weak binding site and also binds to an M-box sequence situated approximately 1.5 or 2.5 helical turns upstream of the Ste11-box (Table 3). The control of P-specific transcription is still an enigma that awaits functional promoter dissection studies to be resolved.

Our observation that the P-specific transcript *map3 *extends into the neighbouring gene *SPAC3F10.09 *demonstrates the importance of mapping the genome-wide location of transcripts and the use of this information in the design of future DNA microarrays. Using a different array system, *SPAC3F10.09 *was reported to be induced in meiosis [9], presumably also reflecting an erroneous detection of *map3 *transcripts transcribed from the opposite strand. Convergent overlapping reading frames are relatively rare in the fission yeast genome, but our finding that the *map3 *transcript extends more than 500 bases downstream of the reading frame suggests that convergent transcripts may overlap even when the reading frames do not.

We also found 92 genes that were more than twofold down regulated by both pheromones (data nor shown). GO categories representing nucleotide and amino acid metabolism were significantly over-represented among these genes, suggesting that specific mechanism exist either to repress these genes or degrade their transcripts during differentiation.

Pheromone communication in fungi was originally considered to be a mechanism by which cells of opposite mating type identified each other, but subsequent studies have demonstrated that it controls many other functions than mere cell recognition. In fission yeast pheromone signalling brings about G_1 _cell-cycle arrest prior to mating, cell elongation towards the partner cell, and is also involved in cell fusion and karyogamy. Finally, it has been known for a decade that entry into meiosis also requires activation of the pheromone pathway. This is because the two meiotic subfunctions *mat1-Pm *and *mat1-Mm *are pheromone controlled; if they are expressed from a heterologous promoter, meiosis is readily activated in the absence of pheromone signalling [7]. Our results in the present study show that – although dispensable for completion of meiosis – pheromone control extends beyond the point of meiotic entry. Thus, several genes required for specific meiotic sub-functions in particular for meiotic recombination turned out being pheromone induced. Consistent with this, expression of the *dmc1 *gene encoding a RecA like helicase was previously shown to be dependent on Ste11 [40]. However, we note that many of these genes do not have optimal Ste11 binding sites in their promoters [see Additional file 1], suggesting that their induction by pheromone may be indirect. Nevertheless, it is evident from our array results that pheromone stimulation in itself can bring about a robust induction of these genes.

Previously, the *bqt1 *gene mediating telomere clustering prior to meiotic horsetail movement was shown to be pheromone induced [41]. These nuclear movements are already initiated in haploid cells challenged with pheromone [42]. We found that *dhc1 *and *dic1*, encoding dynein heavy and intermediate chains respectively were both pheromone induced. Dynein is required for the horsetail movements, and a null mutant in *dhc1 *significantly reduces meiotic recombination [43]. Furthermore, the dynein-associated Ssm4 protein is also pheromone regulated [44] and this study). Taken together, these observations suggest that pheromone signalling orchestrates the entire process of pre-meiotic chromosome movement and associated recombination. It will be interesting to learn whether oscillations in pheromone signalling play a dynamic role in bringing about these processes.

## Conclusion

We have identified 163 pheromone induced genes in fission yeast. Most genes are induced by pheromone in both cell types, and analysis of GO annotations associated with pheromone induction showed that the genes were involved in many aspects of sexual differentiation, including conjugation, and morphological processes associated with mating. Surprisingly, many meiotic gene functions were also pheromone controlled. Our results confirmed that the Ste11 transcription factor controls many pheromone-induced genes. Finally, most cell-type specific genes now appear to be identified in fission yeast.

## Methods

### Strains, growth conditions and RNA preparation

Standard genetic procedures were carried out as described previously [2]. For array experiments we used the strains Eg794 (genotype: *h*^+ ^Δ*mat2,::LEU2 cyr1::ura4*^+ ^*ura4-D18*, [18]) and Eg796 (genotype: *h*^- ^Δ*mat2,3::LEU2 cyr1::ura4*^+ ^*ura4-D18 sxa2*, this study). Cells were grown at 30°C in MSL [45] to a density of 2 × 10^6 ^cells/ml, and chemically synthesized M-factor (made by Schafer-N, Copenhagen, Denmark) or P-factor (made by Alto Bioscience, Birmingham, UK) was added to a final concentration of 1 μg/ml. Samples were taken for RNA preparation after 2, 4 and 6 hours. For validation of the results in *cyr1*^+ ^genetic background cells, we grew the strains Eg544 (genotype: *h*^- ^Δ*mat2,3::LEU2*) and Eg545 (genotype: *h*^+ ^Δ*mat2,3::LEU2*) at 30°C in MSL to a density of 2 × 10^6 ^cells/ml. Cells were collected and resuspended in, respectively MSL, MSL – nitrogen and MSL – nitrogen + the appropriate pheromone (same concentration as above). After 5 hours of incubation, cells were harvested for preparation of RNA.

### Microarray analysis

Total RNA for microarray analysis was prepared as described in [46]. Microarray probe labeling and hybridization was performed as previously described [47], using arrays from Eurogentic SA containing PCR-generated probes in duplicate for 4927 nuclear genes. Slides were scanned using a BIO-RAD Vers-Array ChipReader and quantified with Imagene 4.2 software. Data were normalized by Lowess normalization using GeneSpring software (Agilent Technologies). Temporal expression patterns of genes in response to pheromone treatment were studied using K-means clustering using GeneSpring software (Agilent Technologies). Larger clusters of genes manifesting patterns that were robustly identified independently of cluster number where regarded as significant. Pheromone regulated genes were selected by a variety of criteria including consistent regulation of different samples and replicates by = 2-fold as well as the magnitude of mean fold changes, their significance (one-samole T-test) and variation (SD).

GoMiner [48] was used to identify gene ontology (GO) categories that were significantly (*p *≤ 0.05) enriched in sets of both M-factor and P-factor regulated genes in relation to the frequency of their occurrence in the whole *S. pombe *genome. Microarray data generated in this study are available at Gene Expression Omnibus [49] under accession number GSE5323.

### Prediction of DNA binding sites

The Patser algorithm, available via the Regulatory Site Analysis Tools package [50] was used to search for putative binding sites for the Ste11 transcription factor upstream of the coding regions of genes. The frequency matrix for nucleotides at each position (Table 4) was constructed using sequences found in known Ste11 target genes [14].

The significance of the elevated Ste11-site frequency in pheromone-induced genes was tested in relation to the mean frequency observed in 10 random gene sets of equal size. A one-sample t-test was used to determine whether the mean Ste11-site frequency in the random sets was significantly different from that observed in the sets of pheromone-induced genes. The number of putative Ste11 sites in intergenic regions was also estimated by searching for strings of nucleotides matching the optimal Ste11 site (the inverse sequence of AACAAAGAAA) allowing for 0, 1 or 2 mismatches.

### Real-time and RT PCR analysis

Total RNA was extracted using hot acidic phenol as described above. To remove DNA the RNA samples were digested with DNaseI prior to RT-PCR and real-time PCR analyses. The gene-specific primer sets used for PCR have been described previously [47]. Quantitative real-time PCR reactions were carried out in 96-wells optical reaction plates; using 50 ng DNaseI treated RNA and the QuantiTect SYBR-green RT-PCR kit (Qiagen) supplemented with 10 nM fluorescein. Real-time RT-PCR was developed using the iCycler (BIO-RAD) by single-step amplification. The thermal cycling conditions comprised reverse transcription at 50°C for 30 min and an initial denaturation at 95°C for 15 min, followed by 40 cycles of 95°C for 15 s, 50°C for 30 s and 72°C for 60 s. Each sample was assayed in triplicate. To minimize errors arising from variations in the amount of starting RNA among samples, amplification of *act1 *mRNA served as an internal reference. Standard curves for each relevant gene and for *act1 *were produced by performing serial two-fold dilutions of control RNA (a 1:1 mixture of RNA from pheromone-stimulated cells of opposite mating types). For each experimental sample, the amount of specific transcript was normalized to the amount of *act1 *transcript measured in the same sample.

RT-PCR was carried out using 50 ng DNaseI-treated RNA and the Qiagen OneStep RT-PCR kit. The thermal cycling conditions comprised reverse transcription at 50°C for 30 min and an initial denaturation at 95°C for 15 min, followed by 20–25 cycles of 95°C for 15 s, 50°C for 30 s and 72°C for 60 s. For strand-specific RT-PCR, the reactions were split in two and one of the primers (either sense or anti-sense) was not added until after inactivation of reverse transcriptase.

### Functional characterisation of genes

The open reading frame of *SPBC4.01 *was replaced by the *kmx*-cassette by means of the PCR-based one-step gene disruption procedure, using the oligos OBP421 and ONP422 [51]. The strain deleted for SPAC1565.03 was obtained from Bioneer Corporation, Korea. Sporulation was evaluated by iodine staining as previously described [8].

The 3' end of the *map3 *mRNA was determined by 3' RACE (Rapid Amplification of cDNA Ends). In brief, RNA extracted from pheromone stimulated *P *cells was reverse transcribed using the oligonucleotide SKP62. Next, *map3 *was amplified by PCR using the oligonucleotides SKP63 and ONP441, and the 3' end was identified by sequencing.

Oligonucleotides:

ONP421: TTATAATAGACTTACATTCCATAATAGTTCTTCTACTCCCTAAACTAATT TTGCTAAAGT AAACAATTTAAAATTACGGTTTAAGACGGATCCCCGGTTAATTAA

ONP422: 5' GGTTTATGATTCATTAAAAGCTAATTCTCAATACATTTTTGCTCAAATTAGTAATATGGTTTACAAAATCTTATTTATGTTTGTGAATTCGAGCTCGTTTAAAC

ONP441: 5-ATCAACTTTTCCTTTACTCAATTTA

SKP62: 5'-GACCACGCGTATCGATGTCGACTTTTTTTTTTTTTTTTT

SKP63: 5'-GACCACGCGTATCGATGTCGAC

## Authors' contributions

Y-XF did all microarray experiments including normalization, data processing, identification of enriched GO categories and contributed to the writing process. SK mapped the 3'end of *SPAC3F10.09 *and analysed the mating behaviour of Δ*SPAC1565.03*. CH characterised the Δ*SPBC4.01*phenotype. AW did data mining, analysed the relationship between P-factor and M-factor induced genes, quantiated the Ste11 binding sites and contributed to the writing process. ON did all strain constructions, RNA preps and realtime/RT PCR analyses, and also prepared the manuscript draft. All authors have read and approved the final manuscript.

## Supplementary Material

Additional file 1 Pheromone-induced genes and putative ste11 binding sites in their upstream intergenic regions. See top of Table for column definitions Click here for file

## Figures and Tables

**Table 1:** Examples of genes induced by pheromone.

Gene name:	Systematic name:	Product:	Fold induc. M-factor	Fold induc. P-factor	Reference
agl1	SPAPB24D3.10c	secreted alpha-glucosidase	88.5	19.6	this study
apc2	SPBP23A10.04	anaphase-promoting complex subunit	2.5	1.3	this study
bgl2	SPAC26H5.08c	glucan 1,3-beta-glucosidase	4.7	3.9	this study
bqt1	SPAC6G9.13c	bouquet formation protein	3.8	3.0	[41]
cdc22	SPAC1F7.05	ribonucleoside reductase subunit	4.0	2.7	this study
cdt2	SPAC17H9.19c	activator of ribonucleoside reductase	3.0	1.7	this study
cgs2	SPCC285.09c	cAMP-specific phosphodiesterase	3.4	2.1	this study
cig2	SPAPB2B4.03	S-phase cyclin	5.8	5.7	[52]
cnp1	SPBC1105.17	CENP-A	4.9	2.1	this study
dhc1	SPAC1093.06c	dynein heavy chain	14.9	73.1	[44]
dic1	SPBC646.17c	dynein intermediate chain	9.9	4.6	this study
dmc1	SPAC8E11.03c	RecA family ATPase	11.1	5.9	this study
eme1	SPAPB1E7.06c	Holliday junction resolvase subunit	4.3	2.2	this study
fus1	SPAC20G4.02c	formin	10.7	11.7	[11]
isp6	SPAC4A8.04	vacuolar serine protease	4.0	2.6	this study
mei2	SPAC27D7.03c	RNA-binding meiotic protein	6.7	4.8	this study
mei4	SPBC32H8.11	meiotic forkhead transcription factor	7.6	3.2	this study
mek1	SPAC14C4.03	meiosis specific checkpoint kinase	8.6	4.4	this study
mik1	SPBC660.14	mitotic inhibitor kinase	5.7	4.4	this study
mis4	SPAC31A2.05c	cohesin loading factor	7.4	3.0	this study
mns2	SPAC513.05	alpha-mannosidase	10.9	8.5	this study
msa1	SPAC13G7.13c	RNA-binding protein	4.1	2.4	this study
psm3	SPAC10F6.09c	mitotic cohesin complex subunit	5.4	2.4	this study
rad25	SPAC17A2.13c	14-3-3 protein	2.8	2.1	this study
rad32	SPAC13C5.07	mre11 type nuclease	3.1	1.9	this study
rec10	SPAC25G10.04c	meiotic recombination protein	7.6	4.9	this study
rec12	SPAC17A5.11	Spo11 endonuclease	2.3	3.4	this study
rec25	SPAC17A5.18c	meiotic recombination protein	9.9	9.3	this study
rec27	SPBC577.05c	meiotic recombination protein	2.8	9.3	this study
rep1	SPBC2D10.06	MBF transcription factor subunit	24.0	10.0	[53]
rgs1	SPAC22F3.12c	regulator of Gpa1 signalling	11.7	11.2	[36]
rlc1	SPAC926.03	myosin II regulatory light chain	3.8	3.0	this study
rti1	SPBC119.14	Rad22 homolog	6.4	4.9	this study
sad1	SPBC12D12.01	spindle pole body protein	4.5	2.0	this study
-	SPAP14E8.02	forkhead ass. transcription factor	4.1	2.5	this study
-	SPBC4.01	sequence orphan	115.6	1037.0	this study
spk1	SPAC31G5.09c	pheromone response MAP kinase	12.4	5.8	this study
ssm4	SPAC27D7.13c	p150-Glued protein	3.8	11.3	[44]
ste11	SPBC32C12.02	transcription factor	3.9	3.4	this study
ste4	SPAC1565.04c	adaptor protein	2.8	2.6	this study
ste7	SPAC23E2.03c	meiotic suppressor protein	8.4	7.4	this study
swi5	SPBC409.03	meiotic recombination protein	2.0	3.2	this study
whi5	SPBC800.02	cell cycle transcriptional repressor	3.3	1.4	this study

**Table 2:** Cell-type specific genes in fission yeast

**Gene:**	**Function:**	**Expression pattern:**	**Reference:**
**Genes with a function in M cells**			

*mat1-Mc*	transcription factor	M^1)^	[13]
*mat1-Mm*	activator of meiosis	M^1)^	[7]
*mfm1*	M-factor	M	[12]
*mfm2*	M-factor	M	[12]
*mfm3*	M-factor	M	[12]
*mam1*	M-factor transporter	M	[54]
*mam2*	receptor for P-factor	M	[55]
*mam3*	M cell agglutinin	M^4)^	this study
*mam4*	methylation of M-factor	M,P	[23]
*sxa2*	activator of meiosis	M	[19]
*cwp1*	farnesylation of M-factor	M^2,4)^	this study
*SPAC11H11.03*	not analysed	M^4)^	this study

**Genes with a function in P cells**			

*mat1-Pc*	transcription co-factor	P^1)^	[13]
*mat1-Pm*	activator of meiosis	P^1)^	[56]
*map1*	transcription factor	M,P^3)^	[15, 16]
*map2*	P-factor	P	[57]
*map3*	receptor for M-factor	P	[58]
*map4*	P cell agglutinin	P^4)^	this study
*SPAC1565.03*	unknown	P^4)^	this study

Notes:

^1) ^Activated when transposed to the *mat1 *locus.

^2) ^Expressed at low level in P cells (presumably essential).

^3) ^Also required for stimulation of M-specific gene expression [16].

^4) ^Cell-type specific expression confirmed by time course array experiment.

**Table 3:** Ste11-Box related DNA motifs identified in known and newly discovered M-specific genes

**Gene**	**Strand**	**Start**	**End**	***M/Ste11-Box Motif1^1^***	**Source**
cwp1	D	-175	-134	CATCACACCTGAACAATAGTACGCACTA**AACAAAGA**G**A**C	this study
mam1	R	-161	-122	GTATGGTGGACAACAATGGAGAGTACCT**AACAAAG**GCCC	[14]
mam2^3^	D	-158	-119	GACATAGAAGTGTTTTCTGGAAATTCTA**AACAAAGA**GGA	[14]
mam3	R	-340	-301	TATTTGTCAATTGCAATACTAGTACTAA**AACAAAG**TCTT	this study
mam4^4^	R	-271	-232	AATTATAGTTGACACAATAGGGAAGTAA**AACAAAGAAA**A	this study
mat-Mc^2^	R	-83	-44	AACTACTTACAGAACAAAGAAACCCAAA**AACAAAG**G**AA**C	this study
mat-Mm^2^	D	-90	-51	AACTACTTACAGAACAAAGAAACCCAAA**AACAAAG**G**AA**C	this study
mfm1	D	-191	-152	AACAATTGACTAGACAATGGGTCCGACC**AACAAAGAA**GT	[14]
mfm2	D	-172	-133	AAACAATGGGTCAAACAATAGGCAAATG**AACAAAGA**G**A**T	[14]
mfm3	D	-192	-153	AACAATTAACTAGACAATA000GGCCCAACC**AACAAAGAA**GT	[14]
SPAC11H11.03c^3^	R	-319	-280	GACATAGAAGTGTTTTCTGGAAATTCTA**AACAAAGA**GGA	[14]
sxa2	R	-326	-287	TTTACAATCAACAACAATAGAGATGGGC**AACAAAGA**C**A**C	[14]

M-box sequences are underlined and Ste11 binding sites are given in bold.

^1 ^Motifs were identified in the region 2000 bp upstream of the ATG or in the entire inter-genic region where this is less than 2000 bp

^2 ^Divergently transcribed gene pair

^3 ^Divergently transcribed gene pair

^4 ^Not strictly M-specific. Expression level 2–3 fold higher than that of P cells.

**Table 4:** Sequence frequency matrix used to identify consensus ste11 binding sites

	**Base position**									
	
**Base**	**1**	**2**	**3**	**4**	**5**	**6**	**7**	**8**	**9**	**10**
**A**	2	0	0	0	0	0	0	0	0	0
**C**	2	0	1	17	0	0	0	0	0	0
**G**	0	0	0	0	0	0	0	17	0	0
**T**	13	17	16	0	17	17	17	0	17	17
